# Clinicians in low‐ and middle‐income settings need better access to point‐of‐care haemoglobin tests for identifying and managing children and pregnant women with severe anaemia

**DOI:** 10.1111/tmi.14096

**Published:** 2025-02-23

**Authors:** Annabelle South, Imelda Bates, Sophie Uyoga, Florence Alaroker, Elizabeth C. George

**Affiliations:** ^1^ MRC CTU at UCL Institute of Clinical Trials and Methodology, UCL London UK; ^2^ Centre for Capacity Research Liverpool School of Tropical Medicine Liverpool UK; ^3^ Kenya Medical Research Institute Wellcome Trust Research Programme Kilifi Kenya; ^4^ Paediatrics Soroti Regional Referral Hospital Soroti Uganda

**Keywords:** anaemia, children, haemoglobin, laboratory services, low‐ and middle‐income countries, point‐of‐care testing, pregnancy, universal health coverage

## INTRODUCTION

Around one in four people around the world are affected by anaemia, with 52 million person years lived with disability due to anaemia in 2021 [1]. While anaemia is common around the world, people living in sub‐Saharan Africa and south Asia are most affected, with pregnant women and children bearing the brunt [1]. Severe anaemia can be life‐threatening and requires prompt diagnosis and treatment. The World Health Organization lists automated full blood counts as an essential in vitro diagnostic for use in clinical laboratories, and haemoglobinometers for use in community settings and health facilities without laboratories [2]. In particular, haemoglobin is one of six pathology and laboratory tests that the World Health Organization recommends that all pregnant women should receive [3]. Point‐of‐care (POC) haemoglobin tests can also be useful for urgent clinical care decisions in settings where there is access to laboratories, as they can provide results very quickly. However, access to these important diagnostic tests is limited.

In December 2023, the Medical Research Council Clinical Trials Unit at University College London [4] and Global Health Network [5] hosted a webinar titled ‘Increasing access to timely haemoglobin results: unlocking the potential of point‐of‐care tests’ [6]. The webinar featured researchers and clinicians from Kenya, Uganda, and the United Kingdom, and was attended by more than 200 people from 93 countries. This article summarises the discussion in order to raise awareness of the need for better access to these tests and further research into how to implement these tests in low‐ and middle‐income country (LMIC) settings, which have received little attention to date.

## BENEFITS AND DRAWBACKS OF POC TESTS

POC tests (including electronic devices, colour‐strip tests, and non‐invasive devices) have the potential to enable access to rapid diagnosis, eliminating the need to send samples to laboratories and reducing delays in receiving results. This is particularly useful in settings where laboratories are not nearby or where time is of the essence to inform treatment decisions. They can be carried out by non‐specialist staff who have received appropriate training. Recent years have seen increases in the availability of POC tests for HIV and malaria in primary and secondary care facilities in many countries, allowing people to access appropriate diagnosis and treatment swiftly. However, access to haemoglobin POC tests to identify severe anaemia is lagging behind, even at the secondary care level.

POC tests may also have drawbacks if not used appropriately. Our recent systematic review found some tests have poor diagnostic accuracy in some settings, while others may not function well in some conditions (e.g., where heat or humidity are high), making selection of an appropriate test that will work well in the setting essential. The devices and consumables do have a cost associated with them (as do laboratory tests), and need to be used rationally. As with all diagnostic tests (including laboratory tests), their results must be interpreted with clinical skill and knowledge, including identifying signs of anaemia complications such as breathlessness or shock. Implementation research alongside the roll‐out of these tests can provide evidence of how to use the tests efficiently, including what training is needed for staff, patient acceptability and the cost‐effectiveness of their use. We know the alternative, in the absence of laboratory tests, (diagnosis based on clinical signs alone), is inaccurate and does not make rational use of resources such as blood [7, 8].

## WHY TIMELY AND RELIABLE KNOWLEDGE OF HAEMOGLOBIN LEVELS IS IMPORTANT FOR CHILDREN AND PREGNANT WOMEN

Severe anaemia is particularly prevalent among children under the age of five and women of reproductive age [1]. Identification of those with severe anaemia enables appropriate management and treatment.

For children in particular, time is of the essence – lives may depend on identifying who needs a blood transfusion urgently [9]. In the FEAST trial (for children with shock due to infections), 52% of children with severe anaemia (Hb <5 g/dL) who were not transfused within 8 h of the haemoglobin measurement died, with almost 90% of those deaths occurring within the first 2.5 h [10]. Children who have haemoglobin levels between 4 and 6 g/dL but are clinically stable may not require immediate transfusion, but do need haemoglobin levels to be monitored, as levels can drop, triggering the need for transfusion [9, 11].

Clinical judgement, based on signs and symptoms, is not reliable enough to determine who requires a blood transfusion, and can lead to over‐prescription, wasting scarce resources [7, 8]. Under or over prescription of blood transfusions may also be a problem if a test with insufficient diagnostic accuracy in a particular setting is used. Although the cost of implementing POC haemoglobin tests in a health facility (including buying the devices, consumables and maintenance) is often seen as a barrier to the use of POC haemoglobin tests [12], their use may result in savings to the health system if it prevents over‐prescription of blood transfusion, which is a relatively costly intervention [13, 14] with insufficient supplies of donated blood in many settings [15]. Health economic evaluation of the actual costs and any savings from the use of POC haemoglobin tests in terms of improved health outcomes would be an important part of implementation research on this topic.

## AVAILABILITY OF SUITABLE HAEMOGLOBIN POC TESTS

A recent systematic review found that there were several different POC haemoglobin tests that were suitable and had been successfully used for children in LMIC settings [12]. The review found fairly wide variation in the diagnostic accuracy of different test devices and use in different settings. It states that ‘There is a need for standardised training protocols to reduce errors in sampling technique and interpretation of colour‐based tests’. The review recommended that further research is essential to evaluate training protocols and diagnostic accuracy in real‐world conditions through implementation research and as these tests become routine practice. It concluded that ‘Routine use of POC(Hb)Ts may significantly reduce child mortality in LMICs, where laboratory analysers are often unavailable and anaemia prevalence is high’. Implementation research carried out alongside the roll‐out of these tests would provide the opportunity to strengthen the evidence‐base on acceptability, feasibility, cost and integration into the health care system, and identify approaches to addressing challenges identified in the literature.

While these devices may not be suitable for every context, they are a potentially useful tool. However, these tests are not widely available. Demographic and Health Surveys in 10 LMICs show haemoglobin tests were only available in 14% of basic primary care facilities and in 33% of advanced primary care facilities, considerably lower than the availability of malaria (37% in basic primary care and 77% in advanced primary care) and HIV tests (33% in basic primary care and 72% in advanced primary care) [16, 17]. While over the last two decades, malaria and HIV have seen substantial investments via vertical programmes, anaemia (a cross‐cutting condition) has not received the same support.

## COMPLEMENTARY ROLES FOR LABORATORY AND POC HAEMOGLOBIN TESTS

### Where laboratory tests are unavailable, POC tests increase access to knowledge of haemoglobin levels

POC tests have the potential to complement laboratory full blood counts. Laboratory full blood counts can provide more comprehensive (red blood cell, white blood cell, haemoglobin, platelet counts and haematocrit levels) and accurate information than current POC tests. Often clinicians will be interested in more than just the haemoglobin level of their patients. However, there are many settings where timely laboratory tests are not available. Analysis of data from Demographic and Health Surveys in 10 LMICs found that automatic complete blood counts were only available in around half of hospitals (secondary care level or higher) [17]. Where these laboratory tests are not available at the health facility, POC tests could usefully inform the management of patients with suspected severe anaemia.

This could be particularly valuable in remote rural settings, where the nearest laboratory with the capacity to carry out full blood counts may be a substantial distance away, and in settings where laboratory infrastructure has been damaged by conflict or natural disasters. POC haemoglobin tests may have the potential to help reduce inequalities for people in such areas, allowing their treatment to be informed by timely access to haemoglobin level information, helping to deliver Universal Health Coverage.

### Where laboratory tests are available, POC tests inform urgent clinical management

Another advantage of POC haemoglobin tests is the speed at which results can be obtained—within 2 min of carrying out the test. Where laboratory tests are available, it can often take several hours to receive results [18]—within the context of high mortality within the first 2–3 h for children with severe anaemia, this delay may be costly. Getting an immediate answer in order to inform urgent clinical management may buy time in which a more complete laboratory work‐up can be carried out, to inform ongoing clinical care.

### Where laboratory tests are available, POC tests reduce pressure on stretched laboratory services

Laboratory services in LMIC settings are, in many places, stretched, with a shortfall of diagnostics staff of around 840,000, and current education and training insufficient to maintain even current levels of staffing [16]. In this context, it makes sense to make use of POC haemoglobin tests in situations where the additional information provided by full blood counts is not needed. One example of that is in the follow‐up haemoglobin monitoring recommended as part of the algorithm for management of children with severe anaemia (at 24 and 48 h following initial haemoglobin test for children who receive a blood transfusion, and at these time points plus one at 8 h for children with uncomplicated severe anaemia (Hb 4–6 g/dL) who do not receive an immediate transfusion [9]). This approach would allow appropriate monitoring of children while allowing the limited laboratory capacity to be focused on conducting tests where they add more value. POC haemoglobin tests also have the potential to provide additional checks when there is insufficient quality assurance of laboratory results.

## CONCLUSION

POC haemoglobin tests exist that can swiftly identify people with severe anaemia. These offer a way of providing access to diagnosis for those who are not within easy reach of laboratory services. If used for monitoring those with severe anaemia following initial diagnosis, they may also free up severely limited laboratory capacity for other tests. They can also provide additional checks in the context of insufficient laboratory quality assurance.

The current situation, where POC haemoglobin tests are not available in many primary care facilities in LMICs where they could be particularly valuable, and where laboratory capacity is so stretched, severely impacts the quality of care for children and pregnant women. It may be contributing to health inequalities for those in poor and rural communities. Research into expanding access to these tests must be a priority if we are to address the burden of severe anaemia in these settings and work towards Universal Health Coverage.

## CONFLICT OF INTEREST STATEMENT

The authors declare no conflict of interest.
